# Deciphering the Molecular Mechanism of Incurable Muscle Disease by a Novel Method for the Interpretation of miRNA Dysregulation

**DOI:** 10.3390/ncrna8040048

**Published:** 2022-06-30

**Authors:** David Israeli, Ai Vu Hong, Guillaume Corre, Quentin Miagoux, Isabelle Richard

**Affiliations:** 1 Genethon, 91000 Evry, France; avuhong@genethon.fr (A.V.H.); gcorre@genethon.fr (G.C.); quentin.miag@gmail.com (Q.M.); richard@genethon.fr (I.R.); 2 Université Paris-Saclay, Univ Evry, Inserm, Généthon, Integrare Research Unit UMR_S951, 91000 Evry, France

**Keywords:** Duchenne muscular dystrophy, pathophysiology, miRNA, host gene, bioinformatics, cholesterol, metabolism

## Abstract

It is now well-established that microRNA dysregulation is a hallmark of human diseases, and that aberrant expression of miRNA is not randomly associated with human pathologies but plays a causal role in the pathological process. Investigations of the molecular mechanism that links miRNA dysregulation to pathophysiology can therefore further the understanding of human diseases. The biological effect of miRNA is thought to be mediated principally by miRNA target genes. Consequently, the target genes of dysregulated miRNA serve as a proxy for the biological interpretation of miRNA dysregulation, which is performed by target gene pathway enrichment analysis. However, this method unfortunately often fails to provide testable hypotheses concerning disease mechanisms. In this paper, we describe a method for the interpretation of miRNA dysregulation, which is based on miRNA host genes rather than target genes. Using this approach, we have recently identified the perturbations of lipid metabolism, and cholesterol in particular, in Duchenne muscular dystrophy (DMD). The host gene-based interpretation of miRNA dysregulation therefore represents an attractive alternative method for the biological interpretation of miRNA dysregulation.

## 1. MicroRNA Dysregulation in Human Diseases

MicroRNAs (miRNAs) are regulators of gene expression, which play an important role in many biological processes [1]. An association between miRNA dysregulation and human pathology was initially demonstrated in the field of oncology [2,3], and was later extended to other human diseases [4,5,6,7]. The dysregulation of miRNAs can offer important indications for disease pathophysiology, i.e., the molecular basis of disease. To progress from miRNA dysregulation to a better understanding of the disease mechanism, a critical requirement is a reliable method for the biological interpretation of miRNA dysregulation. MiRNAs are small RNA molecules that mediate gene silencing by guiding Argonaute proteins to target sites that most often reside in the 3′ untranslated region (UTR) of mRNAs. Accordingly, one important central dogma in the field is that miRNAs are exerting their biological activity principally through repression of the expression of their target genes [8,9].

## 2. Problems and Weaknesses of Biological Interpretations of miRNA Dysregulation Using the Target Gene Method

miRNAs are thought to execute their biological activity principally through their target genes. A popular method for the biological interpretation of miRNA dysregulation is therefore based on the prediction of all the target genes, for all the dysregulated miRNAs, and the application of a statistical test for enrichment of pathways associated with the dysregulated miRNAs. However, it has been reported that this method provides a large number of non-specific predictions, with some pathways and terms that are repeatedly associated with many unrelated diseases. In other words, irrespective of the pathology that is studied and of the precise composition of the list of dysregulated miRNAs, the pathway enrichment analysis provides somewhat redundant results.

The methods for the interpretation of miRNA dysregulation, which are based on miRNA target genes, thus fail to provide a specific biological interpretation which, in turn, makes no contribution toward a better understanding of disease mechanisms. The lack of specificity of miRNA dysregulation to enriched pathways may be attributed to two principal causes: (1) individual miRNAs are predicted to target a large number of genes, many of which are, in reality, false positives, and (2) some biological terms are overrepresented in the scientific literature, such as terms related to cancer and stem cells, and these terms are therefore disproportionally linked to miRNA dysregulation.

These issues were highlighted by a number of recent studies. Blaezard et al. reported that miRNA dysregulation in unrelated diseases often predicted the same dozens of significantly enriched functional categories, and similar GO terms are thus recurrent between unrelated diseases [10]. Godard et al. reported that the analysis of several randomly composed lists of miRNAs yielded similar enrichment results, due to a bias toward cancer and cell cycle terms [11]. Further, Pinzón et al. provided evidence that interpretations which are based on target gene predictions are often biologically irrelevant, since many miRNA-predicted target sites may not be sensitive enough to be functionally regulated by the dysregulated miRNA [12]. Finally, it has been demonstrated that target prediction algorithms are oriented toward “model organisms”, whereas a successful prediction rate is reduced in non-model organisms [13].

**Figure 1:**
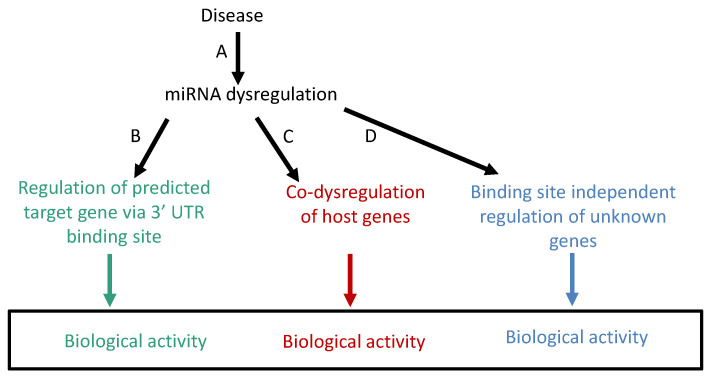
Problems and weaknesses of inferring miRNA biological activity using the target gene method. (A) The analysis of miRNA target genes does not provide information on the causes of miRNA dysregulation because miRNA promoters and expression control mechanisms are insufficiently characterized. (B) Lack of specificity of predicted target genes for dysregulated miRNAs. Predicted target genes are often false positives, which introduces bias into the analysis. (C) Host genes are often co-dysregulated, or part of pathways which are co-dysregulated with their respective hosted miRNAs. Host gene dysregulation may provide important information, not only on the consequences, but also on the causes of dysregulation in the disease state. (D) MiRNAs regulate the expression of genes that are neither target, nor host genes. This dysregulation and its biological interpretation are invisible when using the current analytical methods. Not only are many predicted target genes false positives, in addition, the biological interpretation which is based on the target gene method ignores the biological activity of genes in the two other categories, C and D.

In addition to the lack of specificity, two other important issues may contribute to false, or biased, biological interpretation when the target gene method is used. The first is that the target gene method focuses only on the consequences but ignores the reasons for miRNA dysregulation. However, when investigating a disease, the causes are just as important as the consequences. The second issue is that the biological activity of miRNA is in fact not mediated solely by its target genes. It has recently been shown that a significant number of miRNAs affect cellular and organismal biology via non-canonical activity of miRNA (reviewed in [14,15,16]). This pertains mostly to the activation of gene expression by miRNAs that are located in the nucleus, attached to gene promoters rather than the 3′UTR, and therefore “invisible” to prediction by classical target gene analysis. The problems and weaknesses of inferring miRNA biological activity using the target gene method are illustrated in Figure 1.

## 3. Development of a Complementary Method for the Interpretation of miRNA Dysregulation

In the present commentary, we describe our recent study [17] on the discovery of miRNA dysregulation in the plasma of Duchenne muscular dystrophy (DMD) patients. DMD is the most commonly inherited pediatric muscle disorder. It is a genetic X-linked progressive myopathy, characterized by muscle wasting and weakness, which leads to loss of motor function in puberty, cardiac and respiratory involvement, and premature death. DMD arises due to mutations in the dystrophin gene and occurs at a rate of approximately 1:5000 male births. The disease is caused by a deficiency of dystrophin or the synthesis of functionally impaired dystrophin, a critical protein component of the dystrophin glycoprotein complex, which acts as a link between the cytoskeleton and the extracellular matrix in skeletal and cardiac muscles.

For the interpretation of miRNA dysregulation in DMD, we initially used the classical methodology of miRNA target gene pathway enrichment analysis. In addition, we developed a complementary method for the biological interpretation of miRNA dysregulation, which is based on miRNA host genes, rather than miRNA target genes. In the following paragraphs, we describe the results of the miRNA target gene method, the underlying rationale, and results of the host gene method, and then briefly compare the two approaches.

## 4. Interpretation of miRNA Dysregulation in DMD Using the Target Gene Method

Circulating miRNAs were profiled in the plasma of a relatively large cohort of DMD patients using the high-throughput sequencing method [17], and dysregulated miRNAs were identified. Target genes of dysregulated miRNAs, and their enriched pathways, were predicted by the KEGG function of the DIANA TOOLS miRPath V3 [18], considering all upregulated and downregulated miRNAs (adjusted *p*-value *<* 0.1) in 4–12-year-old DMD patients compared with age-matched controls. The analysis identified a large number of significantly (*p <* 0.05) enriched pathways, including pathways already suspected to be involved in DMD, such as the extracellular matrix (ECM)–receptor interaction, fatty acid biosynthesis, focal adhesion, PI3K-AKT signaling, and Foxo signaling. While agreeing with other data in the field, these results were not specific enough to provide new testable hypotheses concerning DMD pathophysiology. Thus, the outcome of the target gene analysis did not provide further understanding of the disease.

## 5. Interrelations between miRNAs and Their Host Genes

More than half of human miRNAs are embedded within introns and exons of protein-coding genes [19,20]. The embedded miRNAs are often co-expressed with their host genes [21,22], frequently conserved between species [23], and can regulate, directly or not, the expression and activity of their host genes [24,25,26,27,28,29]. These data support strong functional relations between miRNAs, their host genes, and biological activity. By definition, there is only one host gene for any given miRNA, as compared with up to hundreds of potential target genes. MiRNA target gene-based predictions are thus limited by over-complexity and unreliable specificity, while, by definition, miRNA host genes are 100% specific to their hosted miRNA, and likely have a strong functional relationship to their hosted miRNA.

## 6. Co-Dysregulation of miRNA and Host Genes in DMD

Intriguingly, a number of studies did not confirm strong co-expression relationships between miRNAs and their host genes [30,31], which could raise doubts on the strength of miRNA–host gene relationships. However, a careful inspection of the list of dysregulated miRNAs in the serum of DMD patients and their host genes provided us with an important observation. We noticed that 16.9% of the intragenic dysregulated miRNAs (12 miRNAs out of 71) were embedded inside host genes, which are known to be dysregulated in DMD (patients or disease models). Thus, in addition to the possible **co-expression**, this observation pointed to the **co-dysregulation** of miRNAs and their host genes in DMD and provided important support for their functional relationships in a disease state.

## 7. miRNAs and Their Host Genes May Coordinately Regulate Disease State in DMD

**Figure 2:**
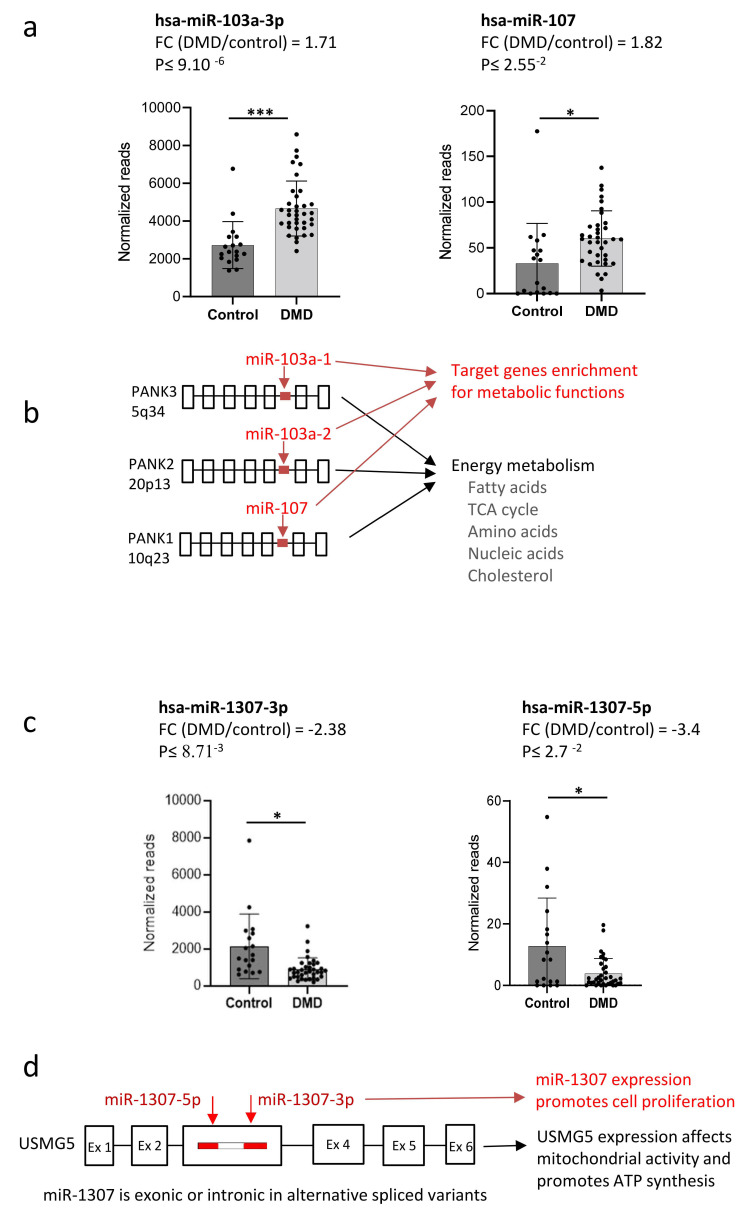
Dysregulated miRNAs may act coordinately with their host genes in DMD. (**a**) miR-103a and miR-107 are dysregulated in DMD patients. MiR-103a-1-3p and -103a-2-3p are indistinguishable by their sequence and are detected and presented together (data taken from [17]). (**b**) MiR-103a-1, -103a-2, and -107 are located in a highly conserved (in vertebrates) position in the *PANK* genes. PANK enzymes catalyze a rate-limiting step in the synthesis of CoA, an essential cofactor for a wide range of energy metabolism reactions. Bioinformatics target gene analysis for miR-103/107 predicts enrichment for metabolic pathways. (**c**) miR-1307 is downregulated in DMD (data taken from [17]). (**d**) miR-1307 is hosted by *USMG5*. Reduced miR-1307 expression may restrict cell proliferation while *USMG5* level may affect ATP synthesis (see main text for more details). P values are indicated as * for *p* < 0.05 and *** for *p* < 0.0005. Gene structure schematic presentations are not in scale.

In the following section, we present two examples of miRNAs and their host genes that may coordinately regulate cellular functions in DMD. In the first example, the three paralog miRNAs, 103a-1, −103a-2, and −107, are upregulated in DMD [17] (Figure 2a), while the expressions of their *PANK* host genes have not been reported to change in the disease state. In the second example, both miR-1307 and its *USMG5* host gene are downregulated in DMD.

In humans, the paralog miR-103a-1, miR-103a-2, and miR-107 reside in a highly conserved intronic position in the *PANK3*, *PANK2*, and *PANK1* genes, respectively [32] (Figure 2b). The *PANK* genes encode for enzymes that catalyze the rate-limiting step in the conversion of pantothenate (vitamin B5) into Coenzyme A (CoA). CoA is essential for a large number of reactions concerning energy metabolism, including pyruvate/lactate conversion, the TCA cycle, and the synthesis of fatty acids, amino acids, and cholesterol [33,34]. Importantly, target genes’ analysis for miRNA 103/107 identified enrichment for metabolic-related genes [32]. Thus, while there is no indication for a direct regulation of the *PANK* genes by their hosted miRNAs, it is conceivable that miR-103 and miR-107 are acting on metabolic pathways coordinately with their *PANK* host genes [32]. Thus, dysregulation of miR-103 and -107 may indicate metabolic adaptation in DMD, not only by the functions of their target genes but also by the coordinated functions of their host genes.

A second example for possible coordinated relations in DMD of a miRNA with its host gene is provided by the tandem miR-1307 and *USMG5*. We previously reported the downregulation of miR-1307-5p and miR-1307-3p in DMD [17] (Figure 2c). miR-1307 resides in the *USMG5* gene (Figure 2d). *USMG5* (encodes for DAPIT) was found to be expressed to a lower level in muscle biopsies of severe as compared to moderate DMD patients [35]. Dapit is involved in the dimerization of the mitochondrial complex 5 ATP synthase, and the knocking down of Dapit resulted in reduced ATP synthesis in Hela cells [36] and in skeletal muscle myotubes [37]. Further, glucocorticoid treatment of the mdx (mouse model for DMD) resulted in recovered Dapit expression, and improved mitochondrial functions [37,38]. The upregulation of miR-1307 is mostly associated with cellular proliferation and carcinogenesis [39,40], and therefore its downregulation in DMD might be linked to reduced cellular proliferation. A hypothesis concerning functional relations between miR-1307 and Dapit in DMD is that a reduced cellular ATP level (due to reduced Dapit expression), concomitantly with a reduced miR-1307 level and de-repression of its target genes, may coordinately restrict cellular proliferation.

**Figure 3:**
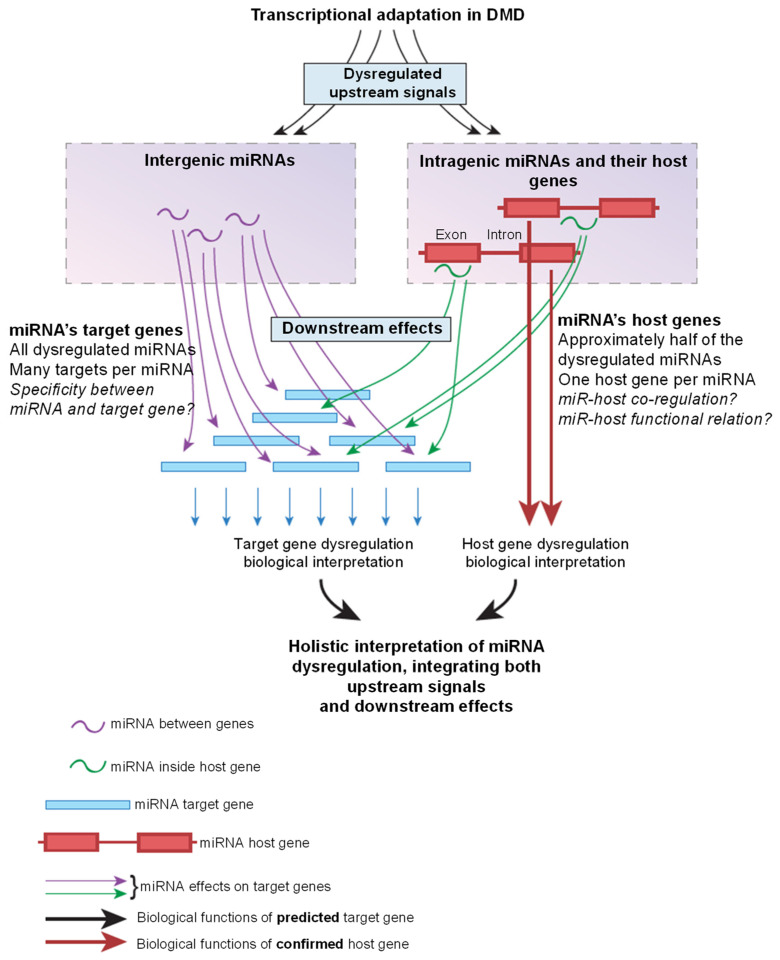
Integrated approach for the biological interpretation of miRNA dysregulation, based on both target and host genes of dysregulated miRNAs. Transcriptional adaptation in the diseased tissue affects both intergenic miRNAs (miRNA between genes, on the (**left**)) and intragenic miRNAs (miRNA inside genes, on the (**right**)). Intragenic miRNAs are often co-dysregulated with their host genes, jointly affecting downstream events. The functional link between a miRNA and its host gene is thought to be strong, while the link between a miRNA and its many predicted target genes is questionable. Dysregulation of intragenic miRNAs and their host genes provides information on upstream signaling in the disease, which causes transcriptional and epigenetic dysregulation, while the analysis of target genes may provide information on downstream events, which are the consequences of miRNA dysregulation.

Based on the co-dysregulation hypothesis, we analyzed pathway enrichment for the host genes of dysregulated miRNA, as an alternative method for the biological interpretation of miRNA dysregulation in DMD. Figure 3 illustrates the approach for the biological interpretation of miRNA dysregulation, based on both target and host genes of the dysregulated miRNAs.

## 8. Interpretation of miRNA Dysregulation in DMD Using the Host Gene Method

miRNA host genes were retrieved from the miRIAD database [19] and manually validated on the Ensembl human genome browser. We considered all miRNAs which are embedded on the sense strand of introns and exons of protein-coding genes. Host genes for a number of different miRNAs in a miRNA cluster were considered only once. All host genes were considered for miRNAs in the same family, which are indistinguishable based on their sequence and are hosted by distinct host genes. This approach resulted in a list of 71 host genes. These 71 genes were subjected to a gene ontology (GO) enrichment analysis using the Ingenuity^®^ Pathway Analysis (IPA^®^, QIAGEN, Venlo, The Netherlands), which resulted in an integrative network in which the SREBP-1 was identified as the most connected molecule. This transcription factor, as well as its family member SREBP-2, was also identified in a central position in the gene network, and these are master regulators of lipid metabolism [41]. A complementary pathway enrichment analysis was performed using ReactomePA and ClusterProfiler R/Bioconductor packages, version 1.40.0 [42]. This complementary analysis suggested the involvement of transport to the Golgi apparatus, transcriptional activity of PPARA and regulation of lipid metabolism, regulation of cholesterol synthesis, and COPI-mediated transport. Thus, in DMD, the SREBP-dependent transcription program, and lipid and cholesterol metabolism, were detected in the center of a network of host genes for dysregulated miRNAs.

## 9. Target Genes versus Host Genes for the Interpretation of miRNA Dysregulation

An important role played by bioinformatics analysis and biological modeling is the capacity to produce new and experimentally testable hypotheses concerning the biological system that is under investigation. As mentioned above, a list of dysregulated miRNAs which was assigned to a target gene enrichment analysis resulted in the identification of many cellular pathways that may mediate pathological pathways in DMD. Five of the twenty most dysregulated pathways are known to participate in the pathophysiology of DMD, while another fifteen (15/20) are only loosely linked to DMD pathophysiology, or not at all. While the five identified DMD-related pathways therefore supported the link between target genes of miRNA dysregulation and DMD pathophysiology, fifteen new propositions for pathway dysregulation made it difficult to formulate a clear and testable hypothesis for the molecular mechanisms of DMD. In sharp contrast, the host gene-based analysis provided one principal prediction: the perturbation of cholesterol metabolism in DMD. The host gene-based method thus outscored the miRNA target gene method in our experimental system, based on the criterion of the capacity to produce a new specific and testable hypothesis.

It is important to note however that the host gene interpretation method is not without weaknesses and limitations. First, only about half of the miRNAs are hosted by host genes, and therefore about half of the data of miRNA dysregulation are non-interpretable by this method. Second, in contrast to the large number of predicted target genes, via the target gene method (and therefore its over-complexity and lack of specificity), the host gene method identifies a relatively small number of genes, to be analyzed for pathway enrichment. To identify a large enough number of host genes for the production of a meaningful pathway enrichment prediction, the host gene method requires a relatively large volume of miRNA expression data, which have to be produced by a large number of biological samples (large cohort), preferentially by the miRNA sequencing method, which may produce greater miRNA dysregulation data, compared to other profiling methods.

## 10. Experimental Validation in DMD

In a perfect scenario, a prediction based on bioinformatics should be validated by experimentation, and a successful experimental validation may provide important support for the pertinence of the bioinformatics method. In a literature survey, we found that the cholesterol-lowering drug, simvastatin, was shown to produce a therapeutic benefit in the mdx mouse model for DMD [43,44]. However, this was attributed to the pleotropic capacity of the drug, rather than its effect on cholesterol metabolism. In our experiment, short-term simvastatin treatment of mdx mice resulted in reduced muscle cholesterol content and improved parameters of the muscle disease. Moreover, an analysis of the cholesterol biosynthesis pathway in the muscle of the treated mice validated the effect of simvastatin on cholesterol synthesis. These results agree with the hypothesis that cholesterol is a potential therapeutic target in DMD. Importantly, these results also provide strong experimental support for the capacity of the host gene method to produce a pertinent biological interpretation of miRNA dysregulation.

## 11. Conclusions

In the present commentary, we described a method for an improved biological interpretation of miRNA dysregulation. The potential of this method was demonstrated based on one example in the domain of muscular dystrophy.

The miRNA host gene analysis outscored the miRNA target gene analysis in its capacity to produce a specific and experimentally testable hypothesis. A surprising prediction of perturbation of cholesterol metabolism was experimentally validated. Meanwhile, a very recent study by another group supports a central role of cholesterol metabolism in DMD [45]. In ongoing investigations by our group, we attempted, with limited success, to translate this discovery into an improved therapeutic protocol in a preclinical model for DMD [46]. Concerning the host gene method for the biological interpretation of miRNA dysregulation, cross-validation of its performance is required by other groups, in other experimental systems.
